# Documentation practice and associated factors among nurses working in public hospitals in Wolaita Zone, Southern Ethiopia

**DOI:** 10.1186/s12912-023-01490-8

**Published:** 2023-09-25

**Authors:** Getachew Nigussie Bolado, Tadele Lankrew Ayalew, Mulualem Gete Feleke, Kirubel Eshetu Haile, Temesgen Geta

**Affiliations:** 1 Department of Nursing, School of Nursing, College of Health Science and Medicine, Wolaita Sodo University, Sodo, Ethiopia; 2 Maternity and Reproductive Health in Nursing, School of Nursing, College of Health Science and Medicine, Wolaita Sodo University, Sodo, Ethiopia

**Keywords:** Nurses, Documentation, Associated factors, Public hospitals, Wolaita Zone

## Abstract

**Background:**

Nursing documentation documents the everyday activities of nursing care that are planned and implemented on individual patients by nurses of different educational statuses. Documentation of nursing activities is the key source of clinical information to meet professional and legal requirements. Although nursing documentation is an important part of nursing practice, it is commonly undone by nurses working with patients for different reasons.

**Objective:**

To assess the documentation practice and their associated factors among nurses working in public hospitals in the Wolaita Zone, Southern Ethiopia.

**Methods:**

An institutional-based cross-sectional study was conducted among 402 nurses and a simple random sampling technique was used to select participants. Data were collected using a pretested structured self-administered questionnaire adapted from previous studies. Statistical Package for the Social Science version 26 was used for data entry and analysis. Independent variables with p-value < 0.25 from bivariable logistic regression were entered into the multivariable logistic regression method and significant associations were obtained at an adjusted odds ratio with a 95% confidence interval and p-value < 0.05.

**Results:**

In this study, the good documentation practice among nurses was 42% [95% confidence interval (CI), 37.2–46.8]. There was a statistically significant relationship between documentation practice and age [adjusted odds ratio (AOR): 2.590 (95% CI: 1.4–4.79)], educational status [AOR: 2.248 (95% CI: 1.13–4.48)], hospital level [AOR: 4.185 (95% CI: 2.63–6.72)], work experience (2–5 years and > 5 years) [AOR: 4.066 (95% CI: 1.55–10.64)] and [AOR: 5.395 (95% CI: 1.97–14.81)] respectively and in-service training [AOR: 0.582 (95% CI: 0.366–0.923)].

**Conclusion and recommendations:**

This study demonstrated that the good practice of documentation among nurses was found to be low. Age, educational status, working in comprehensive specialized hospitals, work experience, and having in-service training had significant associations with documentation practice. It is very important to plan and intervene with different strategies, such as providing training for young nurses, nurses with low educational status, nurses working in primary hospitals, and nurses with less than two years of work experience on documentation standards, to create positive attitudes and enhance their knowledge.

**Supplementary Information:**

The online version contains supplementary material available at 10.1186/s12912-023-01490-8.

## Introduction

Nursing documentation, according to one definition of the term, is a record or chart of nursing care that is organized and provided to individual patients by licensed nurses or other caregivers under the supervision of a qualified nurse [1, 2]. Documentation in nursing is the primary source of clinical information that helps to satisfy legal standards of practice in patient care [3, 4]. Nurses play a vital role in the health care system, and they are having to deal with the documentation process regularly. Nurses having good knowledge about documentation are essential elements in improving patient care. Nursing documentation that is clear, accessible, and accurate is an essential element of quality, safe, and evidence-based nursing care [5, 6]. Nurses practice in all settings at position levels from the bedside to the administrative office; the registered nurse and the advanced practice registered nurse are responsible and accountable for the nursing documentation that is used throughout an organization [5, 7].

Poor documentation of nursing care activities has been shown to have a detrimental effect on healthcare quality [8, 9]. According to a study conducted by the World Health Organization (WHO), poor communication between healthcare workers is a contributing factor to many medical errors. In addition to this, there is evidence linking poor nursing documentation with patient mortality in healthcare institutions [10, 11].

Documentation is the tip of the iceberg of patient care issues that could subject healthcare workers to medical malpractice lawsuits and other types of disciplinary action [11, 12]. Nursing documentation, however, is vital to the effective use of the nursing process for quality nursing care, but it has been observed that nurses often fail to properly document the care they provide, especially related to the use of appropriate nursing terminologies [13, 14].

The documentation practice across different countries and different studies conducted whole over the world. The studies conducted in Nepal, Yemen, Ghana, and Khartoum revealed that 75%, 46.1%, 77.1%, and 69.0% of nurses had good practice of nursing care documentation [1, 15–17]. In Ethiopia, studies were conducted in different parts of the country. The studies revealed that the documentation practice of nursing care ranges from 37.4 to 48.6% [8, 10, 18–23].

The most common barriers and challenges to meeting the documentation expectations were time and resource constraints (i.e. workload), lack of organizational commitment and institutional policies, lack of standards and no single model for health records, lack of professional standards and structures for documentation [14, 15, 17, 24]. Nursing documentation is not well practiced and is reportedly left undone in Ethiopia. The reason for this could be lack of training, lack of time, inadequate understanding, and nurses’ attitudes about nursing documentation [19–21].

In Ethiopia, the Ministry of Health Ethiopia has developed an operational standard for nursing care that outlines that every nursing care provided must be clearly and correctly documented, but the practice among nurses is still poor and lacks quality documentation. To fill this gap, there were limited studies conducted in Ethiopia on nursing documentation [3, 10, 12, 18, 22], but to the best of our knowledge, there is no study conducted in public hospitals of Wolaita Zone. On the other hand, some factors have not yet been studied in Ethiopia and may be associated with the documentation practice of nursing care (level of the hospital and fatigue). In this study, the effect of these factors on the documentation practice among nurses was addressed. Therefore, this study aimed to determine nursing documentation and identify factors associated with it and provide baseline evidence. The findings will help the hospitals to plan different intervention programs to improve poor nursing documentation practice and to tackle factors associated with nursing care documentation.

## Methods and materials

### Study setting and period

This cross-sectional study was carried out among nurses working in selected public hospitals in Wolaita Zone. There are nine public hospitals in this zone, namely: Wolaita Sodo University Comprehensive Specialize Hospital (WSUCSH), Bombe Primary Hospital, Bale Primary Hospital, Gesuba Primary Hospital, Boditi Primary Hospital, Halale Primary Hospital, Bedessa Primary Hospital, Humbo Primary Hospital, and Bitena Primary Hospital. There are 667 permanently employed nurses working in public hospitals included in this study. The data collection period was from November 20 to December 20, 2022.

### Study population

#### Source population

All nurses who were working in Wolaita Zone public hospitals.

#### Study population

All nurses who were working in selected public hospitals in Wolaita Zone were found during the data collection period and fulfilled the inclusion criteria.

### Eligibility criteria

#### Inclusion criteria

Those nurses who had work experience of at least six months were included in the study since nurses with work experience of less than six months or newly employed nurses are not allowed to perform their duty independently and work under the supervision of senior nurses in Ethiopia.

#### Exclusion criteria

Those nurses who were unable to participate in the study due to illness, annual leave, and maternity leave at the time of data collection were excluded from the study.

### Sample size determination

A single population proportion formula was used to determine the sample size, considering the proportion of nursing documentation practice of 47.5% from a study conducted previously in Harari Regional State and Dire Dawa Administration Governmental Hospitals, Eastern Ethiopia [12], with a 5% margin of error and a 95% confidence level as follows.

$$n=\frac{{\left({Z}_{1-\raisebox{1ex}{$\alpha $}\!\left/ \!\raisebox{-1ex}{$2$}\right.}\right)}^{2}P\left(1-P\right)}{{d}^{2}}$$

$$n=\frac{\left(1.96\right)2. \left(0.475 x 0.525\right)}{{0.05}^{2}}=383$$

Where.

n = estimated sample size.

Z = Confidence level (alpha, α).

P = proportion/magnitude of nurses’ documentation practice.

d = marginal error.

**Fig. 1:**
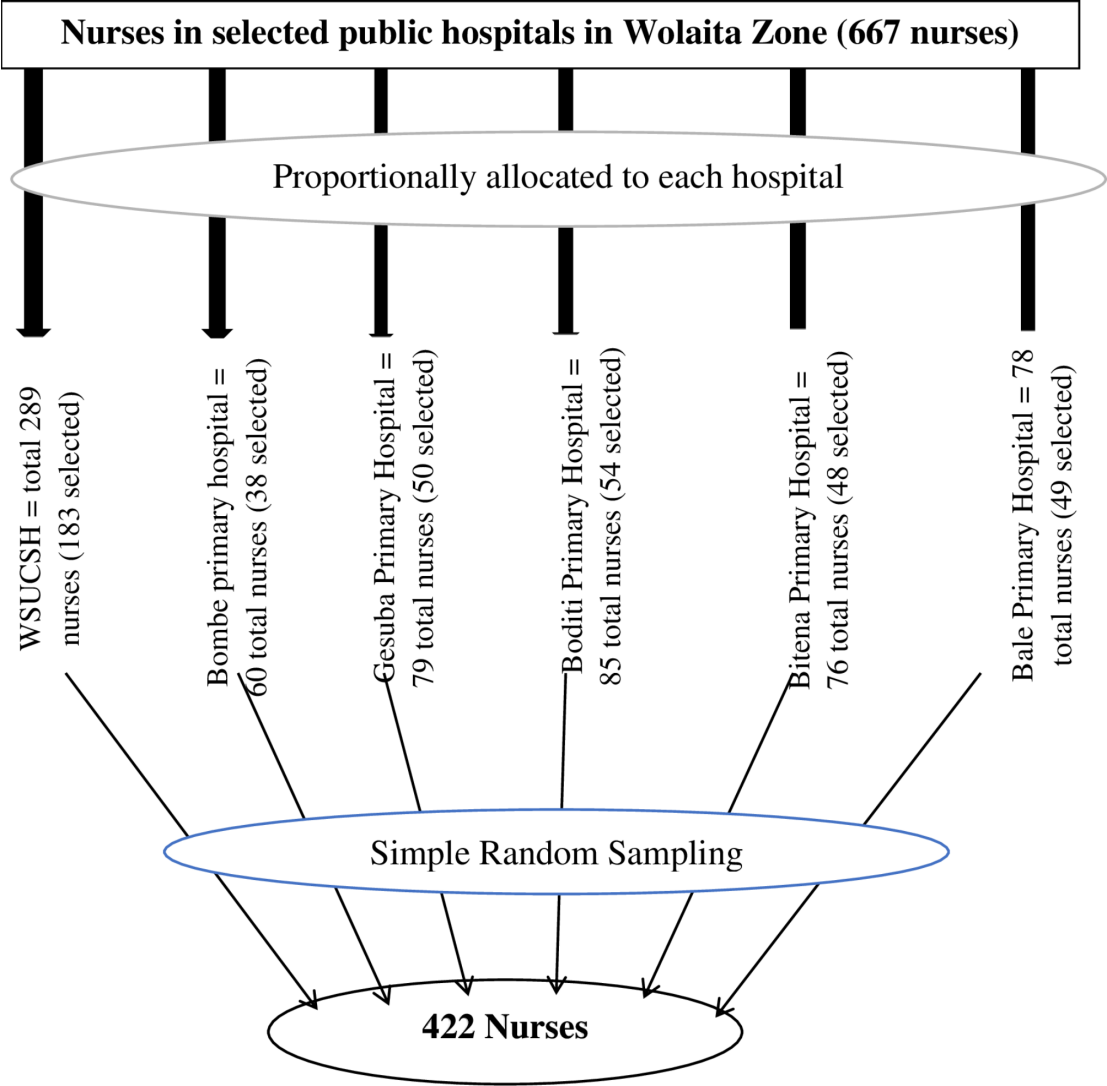
Schematic presentation of sampling procedure in selected public hospitals of the Wolaita Zone, Southern Ethiopia, 2022 (n = 402)

After adding a 10% non-response rate, the final sample size was 422. Among the nine public hospitals in Wolaita Zone, six hospitals were selected using a simple random sampling technique, and there were 667 permanently employed nurses working at these hospitals during the study period. A proportional allocation was conducted for each hospital based on the actual number of nurses. The proportionate number of study participants was determined by using n = nf/N*ni, where ni = the number of nurses in each hospital, nf = the total sample size, and N = the total number of nurses working in selected public hospitals in Wolaita Zone (Fig. 1). Finally, a simple random sampling technique using a lottery method was used to select the participants by using their payroll lists taken from the human resources office of each hospital as a sampling frame.

### Data collection tool and technique

A structured and pretested self-administered questionnaire which was adapted from previous studies conducted in Ethiopia was used to collect data regarding nursing documentation practice and associated factors [10, 20]. The questionnaires were divided into 5 sub-sections. The first part of the questionnaire contains items for sociodemographic characteristics [3, 8, 10, 12, 18, 20, 22]. The second part of the questionnaire contains questions assessing the practice of nurses on documentation [8, 12, 20]. The third and fourth parts of the questionnaire contained questions concerning the knowledge of nurses on documentation and questions assessing the attitude of respondents towards nursing documentation respectively [8, 10, 12, 20, 22, 23]. The final part of the questionnaire contains items assessing organizational factors [12, 15, 18, 22].

### Study variables

#### Dependent variable

Practice of nursing care documentation.

#### Independent variables

Sociodemographic characteristics (age, gender, marital status, educational status, professional category, level of hospital, working unit, experience, and income).

Nurse-related factors (knowledge of nursing care documentation, Attitude toward nursing care documentation,)

Organizational factors affecting the nursing care documentation (nurse-to-patient ratio, availability of documenting sheet, time adequacy, familiarity with documentation guidelines, availability of obligation from the hospital, motivation from supervisors, in-service training, lack of skill, etc.)

### Operational definition

#### Nursing documentation practice

The nurses’ self-reported practice of recording written by nurses and the total written information concerning a patient’s health status, nursing needs, nursing care, and response to nursing care. that was measured by using a structured self -administered questionnaire containing 9 questions which are multiple choice type questions with “Never”, “Sometimes”, and “Always” responses having a score of “1”, “2”, and “3” respectively. This questionnaire has Cronbach’s alpha of 0. 888. Then the core was categorized as good documentation practice and poor documentation practice [10, 12].

#### Good documentation practice

Those respondents who scored above or equal to the mean score of practice questions [10, 12].

#### Poor documentation practice

Those respondents who scored below the mean score of practice questions [10, 12].

#### Knowledge of nursing documentation

it was measured by using 10 items with multiple response type questions. The items had “yes” and “no” responses with a score of 1 given for the correct response and zero for the incorrect response. Finally, those who scored greater than or equal to 55% of correct answers were categorized as having good knowledge and those who scored less than 55% of correct answers were categorized as having poor knowledge of nursing care documentation [8, 19].

#### Attitude towards nursing documentation

There were 10 items used to measure the attitude of study participants towards nursing documentation which had a five-point Likert scale with which 1 denoting “strongly disagree” and 5 denoting “strongly agree”. Those participants who scores greater than or equal to the mean score of total attitude questions were categorized as having favorable attitudes and those who scored less than the mean score of total attitude questions were categorized as having unfavorable attitudes [8, 19].

#### Adequate nurse-to-patient ratio

When a nurse serves ≤ 2 patients in Intensive Care Unit (ICU) or ≤ 6 patients in other than ICU [19].

### Data processing and analysis

The collected data were entered into Statistical Package for the Social Sciences (SPSS) Version 26 for analysis. Descriptive statistics such as tables, graphs, frequencies, and percentages were used to describe the sample characteristics. The bivariable and multivariable logistic regression method was used to find the association between dependent and independent variables. All independent variables with a p-value less than 0.25 from the bivariable logistic regression model were entered into the multivariable logistic regression model. A significant association was obtained at an adjusted odds ratio (AOR) with a 95% confidence interval (CI) and p-value less than 0.05 for interpretation.

### Data quality control

A pre-test of the questionnaire was conducted in Durame primary hospital which is out of the target hospitals on 5% of the sample size a week before the actual data collection period and necessary amendments were done such as unclear questions, typing errors, and ambiguous words accordingly. One day training was given to data collectors on the data collection tool and how to conduct the collection. The process of data collection was supervised by the principal investigator. The principal investigator also checked the completeness, accuracy, and consistency of collected data every day. Double data entry method by two data clerks and the consistencies of the entered data were cross-checked by comparing the two separately entered data on SPSS.

## Results

### Sociodemographic characteristics of participants

Out of 422 study participants, 402 participated in this study, yielding a response rate of 95.3%. Among the participants, 242 (60.2%) were males, and the mean age of the respondents was 33 years (standard deviation: 8.8). About 254 (63.2%) of the respondents were married, and more than half 215 (53.5%) of the study participants had an educational status of bachelor’s degree. Two hundred twenty-nine (57.0%) of the participants were working in primary hospitals, while the rest were working in comprehensive specialized hospitals, and 169 (42.1%) were general or comprehensive nurses. In terms of work experience, 189 (47.0%) of participants have at least 5 years of experience, and the average monthly salary of respondents was 5677.00 birr (standard deviation: 1545.00) (Table 1).

**Table 1:** Sociodemographic characteristics of nurses working in selected public hospitals in the Wolaita Zone, Southern Ethiopia, 2022 (n = 402)

Variable Name	Category	Frequency(n)	Percentage (%)
Age in years	20–29 years	172	42.8
	30–39 years	136	33.8
	≥ 40 years	94	23.4
Gender	Male	242	60.2
	Female	160	39.8
Marital status	Single	130	32.3
	Married	254	63.2
	Others	18	4.5
Educational status	Diploma	132	32.8
	First degree	215	53.5
	s degree and above	55	13.7
Professional category	General/comprehensive nurse	169	42.1
	Emergency Nurse	89	22.1
	Surgical and operation theatre nurse	46	11.4
	Pediatric nurse	52	12.9
	Adult health nurse	28	7.0
	Others*	18	4.5
Level of hospital	Comprehensive specialized hospital	173	43.0
	Primary hospital	229	57.0
Working unit/Ward	Medical ward	89	22.1
	Surgical ward	77	19.2
	Pediatric ward	56	13.9
	Emergency ward	66	16.4
	Outpatient department	44	10.9
	Orthopedics ward	38	9.5
	Others**	32	8.0
Experience	< 2 years	42	10.5
	2–5 years	171	42.5
	> 5 years	189	47.0
Monthly salary	≤ 4085 ETB	70	17.4
	4086–5294 ETB	79	16.7
	≥ 5295 ETB	253	62.9

ETB: Ethiopian Birr, Others*= Neonatal nurse, Oncology nurse, Psychiatry nurse, Ophthalmic nurse. Others**=Intensive care unit, Dialysis center, and nurses on administrative positions

### Documentation practice among nurses

**Fig. 2:**
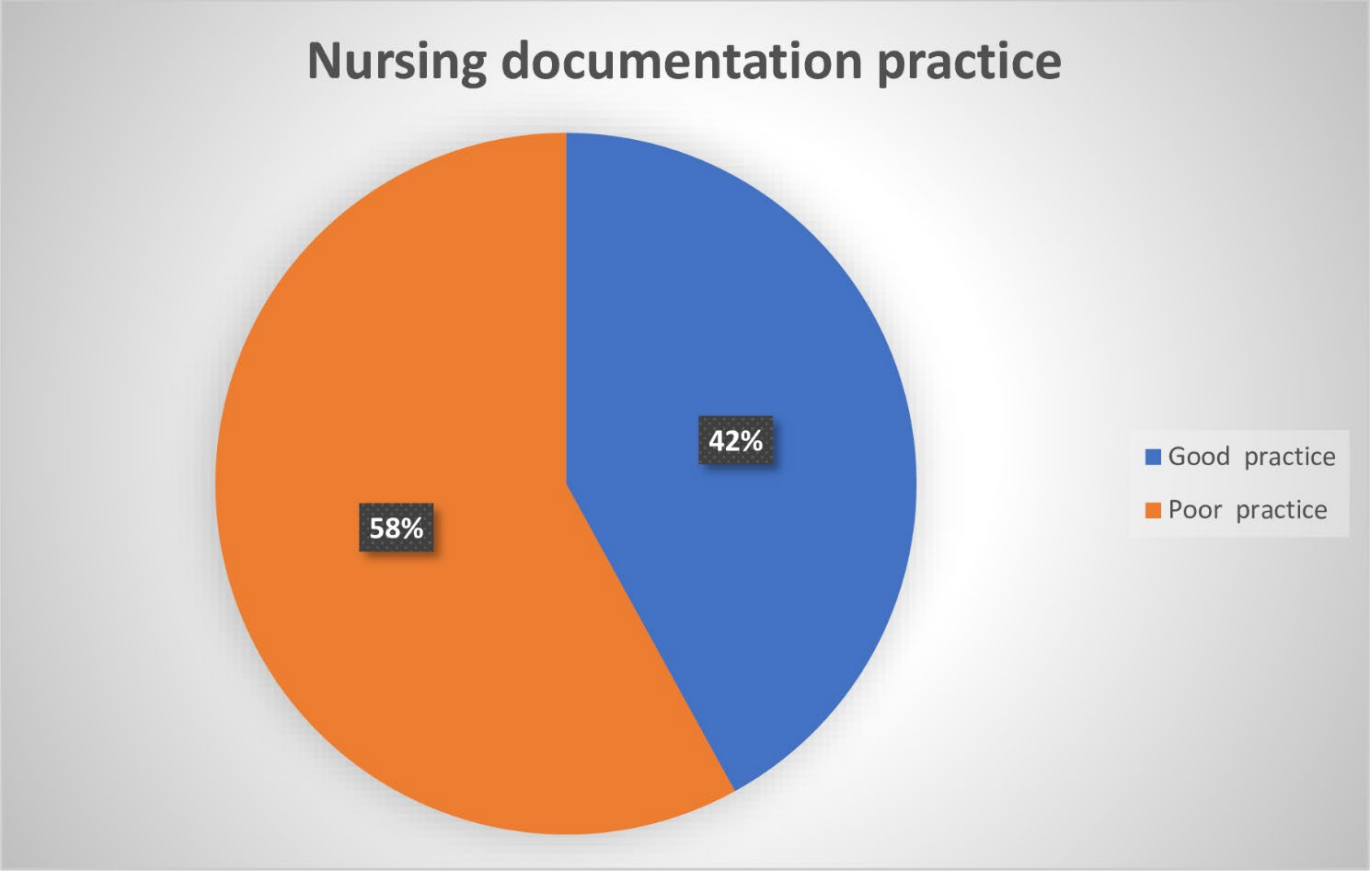
Documentation practice among nurses working in selected public hospitals of the Wolaita Zone, Southern Ethiopia, 2022 (n = 402)

Out of 402 nurses who participated in the study and worked in selected public hospitals in the Wolaita Zone, 169 (42%) [95% CI of37.2 to 46.8] had good nursing documentation practice, whereas the rest 56% (n = 217) had poor nursing documentation practice (Fig. 2).

### Knowledge of nurses about nursing documentation

**Fig. 3:**
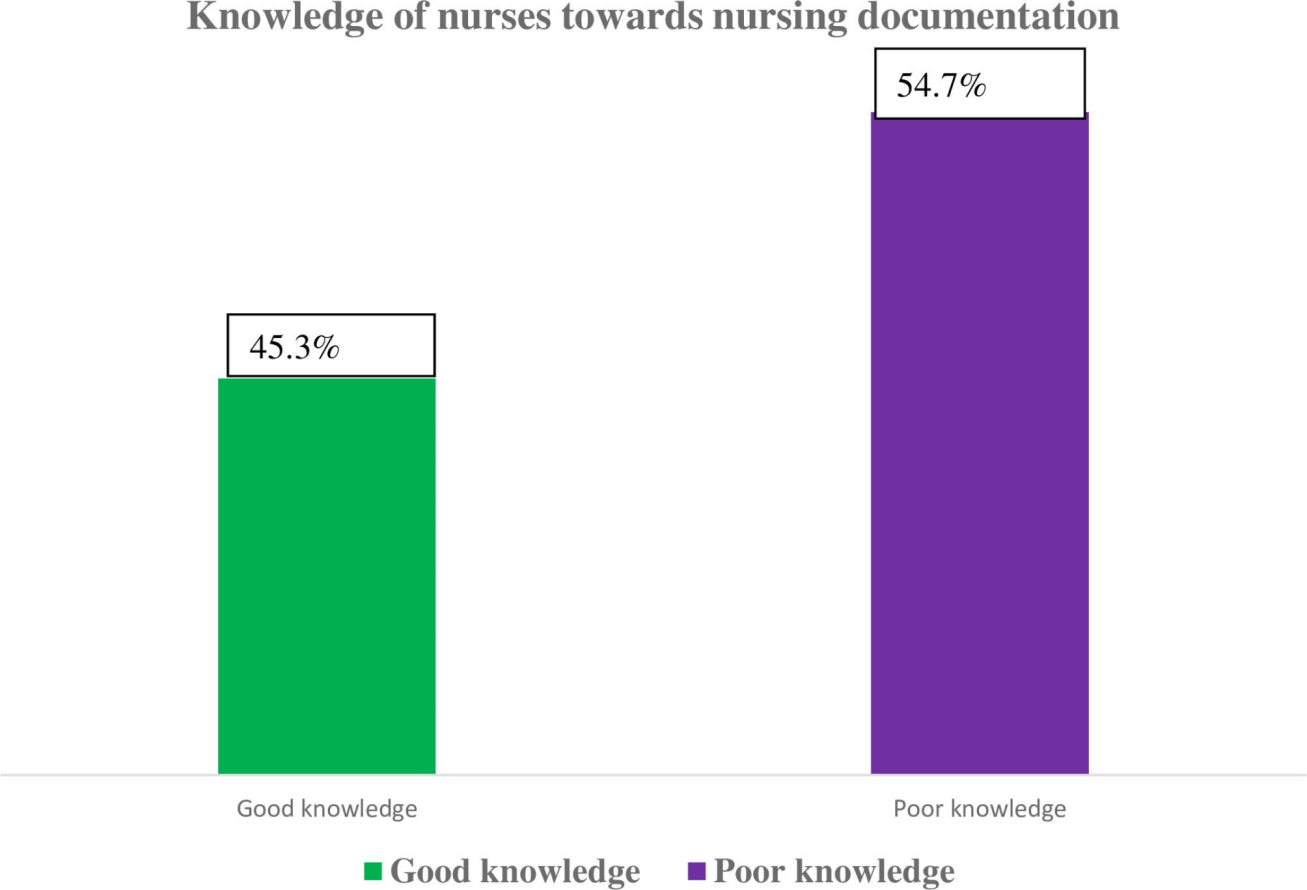
Knowledge of nurses about nursing documentation in selected public hospitals of the Wolaita Zone, Southern Ethiopia, 2022 (n = 402)

The mean score of knowledge items scored by study participants was 23.1 (standard deviation: 2.43). Based on this, the knowledge of nurses about nursing documentation was dichotomized into good and poor knowledge of nursing documentation. Among the participants, 182 (45.5%) had good knowledge, while the rest of 220 (54.7%) had poor knowledge about nursing documentation (Fig. 3).

### The attitude of nurses toward nursing documentation

**Fig. 4:**
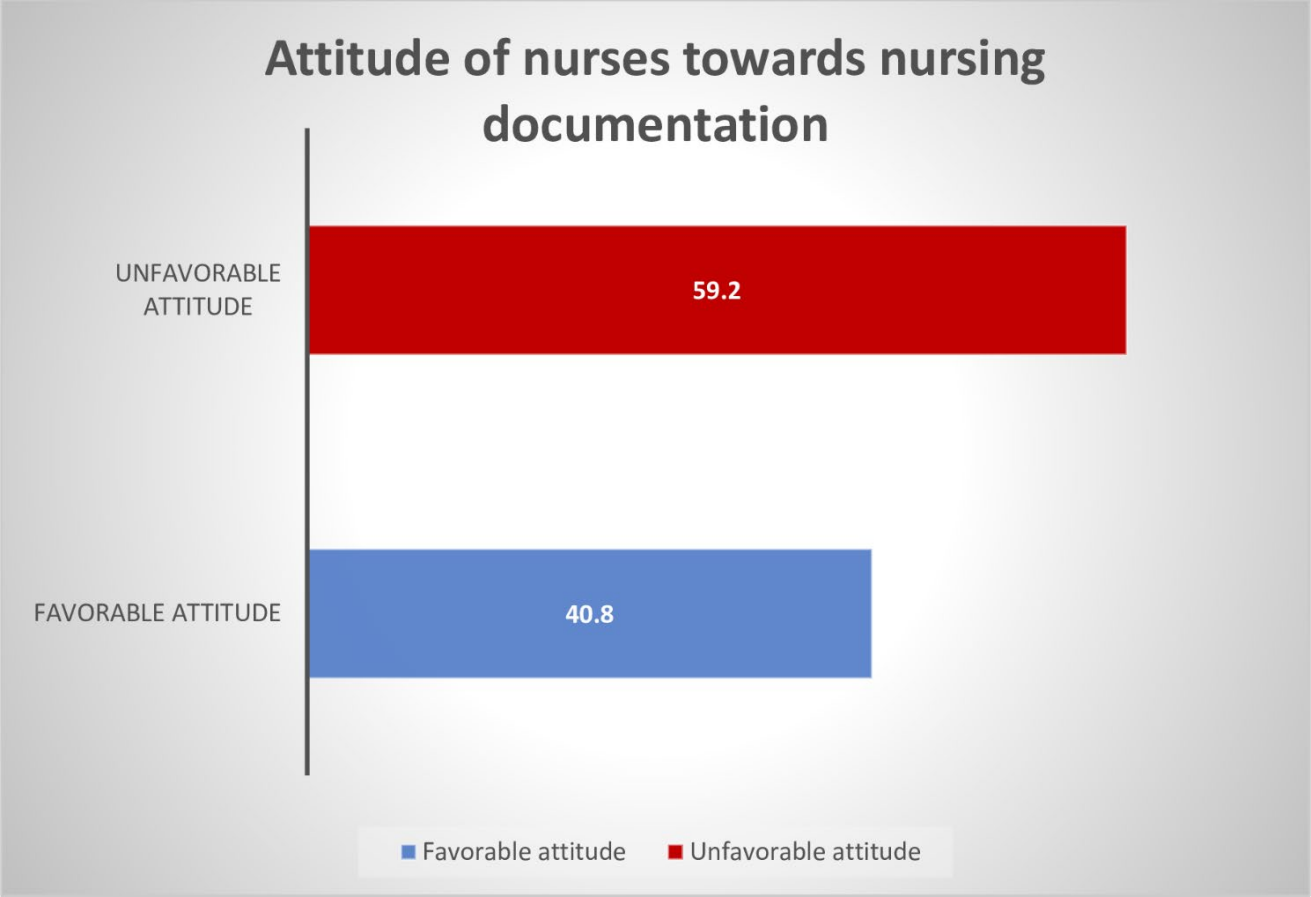
Attitude of nurses towards documentation in selected public hospitals of the Wolaita Zone, Southern Ethiopia, 2022 (n = 402)

This study has revealed that 164 (40.8%) of the respondents had a favorable attitude toward nursing documentation (Fig. 4). Among the participants, 335 (83.3%) responded that they document the assessments they have done for every patient, and 287 (71.4%) said that they document problems or nursing diagnoses they find for every patient. Two hundred fifty-one (62.4%) respondents said that documenting the care given to patients right away is important for both the patient and the nurse.

### Organizational factors influencing nursing documentation

Among the participants, half of them, 202 (50.2%,) got in-service training on standard nursing care documentation, and 191 (47.5%) reported that they have enough time for nursing documentation. Of the respondents, 199 (49.5%) and 247 (61.4%) reported that they have been informed of the availability of operational standards for nursing documentation and the availability of enough sheets for documentation in hospitals. Similarly, of the respondents, 289 (70.1%) said that they were not familiar with the operational standard of documentation, and 330 (79.6%) of them reported that they hadn’t received any motivation or support from the supervisor of their hospitals. Among the participants, 232 (57.7%) responded that there was no monitoring and evaluation system for nursing documentation in their hospitals, and similarly, 233 (58.0%) and 235 (58.5%) reported that there is inadequate staffing of nurses and they do not feel fatigue during patient care that prevents them from documenting nursing care activities (Table 2).

**Table 2:** Organizational factors affecting documentation practice among nurses working in selected public hospitals of the Wolaita Zone, Southern Ethiopia, 2022 (n = 402)

Variables		Frequency (n)	Percentage (%)
In-service training	Yes	202	50.2
	No	200	49.8
Time shortage for documentation	Yes	191	47.5
	No	211	52.5
Availability of operational standard for nursing documentation	Yes	199	49.5
	No	203	50.5
Familiarity with operational Standard	Yes	120	29.9
	No	282	70.1
Availability of sheet for documentation	Yes	247	61.4
	No	155	38.6
Motivation from a supervisor	Yes	82	20.4
	No	320	79.6
Availability of obligation from the hospital	Yes	175	43.5
	No	227	56.5
Availability of monitoring and evaluation	Yes	170	42.3
	No	232	57.7
Staff adequacy	Yes	169	42.0
	No	233	58.0
Fatigue	Yes	167	41.5
	No	235	58.5

### Factors Associated with nursing documentation practice

Bivariable and multivariable logistic regression was done, and age, educational status, hospital level, work experience, and training showed statistically significant associations with nursing documentation practice. Nurses who were aged above 40 years were 2.59 times more likely to practice nursing documentation than those who were aged between 20 and 29 years [AOR: 2.590 (95% CI: 1.4–4.79)]. Nurses who were master’s degree holders were 2.25 times more likely to document nursing care as compared with those who had the educational status of diploma holders [AOR: 2.248 (95% CI: 1.13–4.48)]. On the other hand, those nurses working in comprehensive specialized hospitals were 4.2 times more likely to document nursing care than those who were working in primary hospitals [AOR: 4.185 (95% CI: 2.63—6.72)]. In terms of work experience, nurses with 2–5 years of experience and those with more than 5 years of experience were 4.1 and 5.34 times more likely to practice nursing care documentation, respectively, than those with less than 2 years of experience [AOR: 4.066 (95% CI: 1.55–10.64)] and [AOR: 5.395 (95% CI: 1.97–14.81)]. From organizational factors, in-service training was a statistically significant association with nursing documentation practice. Nurses who hadn’t gotten in-service training were 40% less likely to practice nursing documentation than those who had gotten in-service training [AOR: 0.582 (95% CI: 0.366–0.923)] (Table 3).

**Table 3:** Bivariable and multivariable binary logistic regression analysis on factors associated with documentation practice among nurses working in selected public hospitals in the Wolaita Zone, Southern Ethiopia, 2022 (n = 402)

Variables	Documentation practice		COR (95% CI)	AOR (95% CI)	p-value
	Good n (%)	Poor n (%)			
Age					
20–29	53	119	1	1	
30–39	64	72	1.99		
≥ 40	52	42	2.78 (1.65–4.67)	2.57 (1.4–4.8)	0.002
Educational status					
Diploma	37	95	1	1	
Bachelor degree	104	111	2.41 (1.51–3.83)		
Master degree	28	27	2.66 (1.39–5.11)	2.25 (1.13–4.48)	0.021
Hospital level					
CSH	101	72	3.32 (2.19–5.02)	4.19 (2.63–6.67)	< 0.001
Primary hospital	68	161	1	1	
Work experience					
< 2 years	8	34	1	1	
2–5 years	64	107	2.54 (1.11–5.83)	4.07 (1.55–10.64)	0.004
> 5 years	97	92	4.48 (1.97–10.19)	5.39 (1.97–14.81)	0.001
In-service training					
No	78	124	0.75 (0.51–1.12)	0.582 (0.37–0.92)	0.022
Yes	91	109	1	1	

## Discussion

This study aimed to determine the nursing documentation practice of nurses working in selected public hospitals of Wolaita Zone. It also tried to identify factors that were associated with nursing documentation practice. According to this study, the good documentation practice among nurses was 42% [95% CI of 37.2–46.8%]. This finding was in line with the studies conducted at the University of Gondar Teaching Hospital on physicians’ and nurses’ documentation practice (46.8%) [21], the unfinished task of nursing care in the University of Gondar Hospital (37.4) [22] and Yemen (46.1%) [17].

However, this finding was lower than findings of the studies in North Shoa (47.7%) [8], private hospitals in the Amhara region (47.2%) [20], Harari Regional State and Dire Dawa Administration Governmental Hospitals (47.5%) [12], West Gojjam Zone public hospitals (47.5%) [23], Public Hospitals in Addis Ababa (47.8%) [10], public hospitals in Tigray (47.8%) [3], Jimma University Medical Center (48.6%) [18], Ghana (77.1%) [15], Khartoum (69.0%) [16], and Nepal (75%) [1]. This discrepancy might be due to the differences in study areas (some studies were at single sites, but our study used multiple sites with both inpatient and outpatient departments), study time gaps, measurement tools (for instance, the study conducted in Harari Regional State and Dire Dawa Administration Governmental Hospitals used both self-administered questionnaires and medical record review), and differences in sample sizes. Similarly, it might be due to differences in knowledge and attitudes of nurses towards nursing documentation practice [18], lack of in-service training about documentation [19], and nurses who participated in the above studies might be more familiar with documentation guidelines available in their hospital [10, 18]. The other reason could be differences in the level of hospitals included in this study (there was one comprehensive specialized hospital and six primary hospitals included in this study), while other studies used all specialized hospitals [10]. This incongruence might be because of a high workload, inadequate staff nurses, a lack of time, an unfavorable working environment, and organizational structure (3, 19), as well as low attention to the needs of nurses or a lack of or limited support from hospital management and leaders. The first significant factor associated with nursing documentation practice was the age of participants. Nurses who were aged above 40 years were 2.59 times more likely to practice nursing documentation than those who were aged between 20 and 29 years. This finding was consistent with the studies conducted in private hospitals in the Amhara region [20], Harari regional state and Dire Dawa administration governmental hospitals [12], and Ghana [15]. This might be because the majority of elderly nursing professionals have long time service histories as indicated by the data, their exposure to training opportunities might be expanded, which would familiarize them with operational guidelines for nursing documentation. Once more, as individuals get older, they may recognize many documentation benefits on a variety of levels, which will improve their attitude toward documenting and, in turn, their documentation practices. It is crucial to plan and conduct experience exchanges about documenting practices both inside and between hospitals.

The educational status of nurses was also significantly associated with nursing documentation practice. Nurses who were master’s degree holders were 2.25 times more likely to document nursing care as compared with those who had the educational status of diploma holders. This finding was in line with the studies conducted in Ghana [15] and Nepal [1]. This might be due to the reason that nurses with high educational status (i.e. master’s degree and above), and knowledge of documentation tend to possess better knowledge, and attitudes towards nursing documentation increase. The work experience of nurses had a positive association with documentation practice. Nurses with 2–5 years of experience and those with more than 5 years of experience were 4.1 and 5.34 times more likely to practice nursing care documentation, respectively than those with less than 2 years of experience .This is in line with studies conducted in West Gojjam [23], Ghana [15], and Yemen [17]. The possible reason for this similarity might be that more experienced nurses had a favorable attitude toward nursing care documentation. As nurses’ work experience increases, they face many challenges, including legal suits due to the impacts of poor nursing documentation, so their attitude about the importance of nursing documentation improves over time.

The other variable significantly associated with the documentation practice of nurses is the level of the hospital they are working in. Those nurses working in comprehensive specialized hospitals were 4.2 times more likely to document nursing care than those who were working in primary hospitals This might be because the resources used for documentation, such as documentation sheets, might be more available in comprehensive specialized hospitals than primary hospitals. This might also be due to more in-service training opportunities than in primary hospitals because most of the comprehensive specialized hospitals in Ethiopia are owned by governmental universities, so they can easily provide in-service training opportunities for their healthcare workers, including nurses, in collaboration with governmental and non-governmental organizations in Ethiopia and abroad. This can also improve the practice of documentation among nurses.

Similarly, in-service training was another factor associated with the practice of documentation among nurses. This result is analogous to the outcomes of research carried out in Jimma [18], private hospitals in Amhara region [20], University of Gondar [22], Harar and Dire Dawa [12], Khartoum [16], and Ghana [15]. The reason for this congruency might be a result of in-service training, which may improve their attitude toward documentation, increase their familiarity with operational requirements for documentation, and also boost the value they place on documenting their work. Therefore, training will be useful in enhancing nursing documentation practices.

### Strengths and limitations of the study

The study was conducted with an optimum sample size, and more than two-thirds of the public hospitals in the Wolaita Zone were included. The major limitation of this study was the cross-sectional nature of the study design, which does not show a cause-and-effect relationship between dependent and explanatory variables. Another limitation of this study was that the data were collected through a self-administered questionnaire, which might be subject to response bias from the respondents.

## Conclusion

This study assessed the documentation practice among nurses and its associated factors. Generally, more than half of the nurses working in the public hospitals of the Wolaita Zone had poor documentation practice. Age, educational status, working in comprehensive specialized hospitals, work experience and having in-service training had statistically significant associations with documentation practices.

## Recommendations

Attention should be given to factors that affect the documentation practice among nurses, and there should be designed programs to give in-service training, increase the supply of necessary resources such as documentation sheets, especially for primary hospitals, and provide regular follow-up and interventions to improve the documentation practice. Upcoming researchers are recommended to study the nursing documentation practice using an observational study design to evaluate the generalizability of the findings.

### Electronic supplementary material

Below is the link to the electronic supplementary material.

Supplementary Material 1

## Data Availability

The datasets used and/or analyzed during the current study are available from the corresponding author upon reasonable request.

## Abbreviations

AOR: Adjusted Odds Ratio
CSH: Comprehensive Specialized Hospital
CI: Confidence Interval
ETB: Ethiopian Birr
ICU: Intensive Care Unit
SPSS: Statistical Package for Social Science
WHO: World Health Organization
WSUCSH: Wolaita Sodo University Comprehensive Specialized Hospital
