# Supplementary material for: Documentation practice and associated factors among nurses working in public hospitals in Wolaita Zone, Southern Ethiopia
Source: BMC Nurs. 2023 Sep 25;22:330. doi: 10.1186/s12912-023-01490-8 (PMC10518942; doi:10.1186/s12912-023-01490-8)
Supplement: Supplementary file 1 — Supplementary Material 1 [file 12912_2023_1490_MOESM1_ESM.docx]

**Section I: Sociodemographic characteristics of the nurses working in public hospitals in Wolaita Zone, Southern Ethiopia, 2022**

| SN | Question | Response | Remark |
| --- | --- | --- | --- |
|  | Your age (in years) | Your age (in years) |  |
|  | Gender | 1. Male 2. Female |  |
|  | Marital status | 1. Single 2. Married 3. Widowed 4. Divorced |  |
|  | Your educational status | 1. Diploma 2. First degree 3. Second-degree and above |  |
|  | What is your professional category? | General/comprehensive nurse  Emergency Nurse  Surgical and operation theatre nurse  Pediatric nurse  Adult health nurse  Others |  |
|  | Level of hospital you are working now? | Comprehensive specialized hospital  Primary hospital |  |
|  | Working unit/Ward you are working? | Medical ward  Surgical ward  Pediatric ward  Emergency ward  Outpatient department  Orthopedics ward  Others |  |
|  | Experience (in years) | _____ |  |
|  | You monthly salary | _______ETB |  |

**Section II- Practice of documentation among nurses working in public hospitals in Wolaita Zone, Southern Ethiopia, 2022**

***Instruction-Please read the following questions carefully and put (√) on the correct answer option.***

| **SN** | **Items** | **Never** | **Sometimes** | **Always** | **Remark** |
| --- | --- | --- | --- | --- | --- |
| 1 | Do you document the assessments you have done for every patient? |  |  |  |  |
| 2 | Do you document problems you find (the nursing diagnosis) for every patient? |  |  |  |  |
| 3 | Do you document the intervention you have done for every patient? |  |  |  |  |
| 4 | Do you document the response to your intervention for every patient? |  |  |  |  |
| 5 | Do you document the fluid you administered to every patient? |  |  |  |  |
| 6 | Do you document the medication you administered to every patent? |  |  |  |  |
| 7 | Do you document the education or advice you have provided to a patient? |  |  |  |  |
| 8 | Do you document the fluid balance status of the patient? |  |  |  |  |
| 9 | Is all your documentation done immediately after care provided to the patient? |  |  |  |  |

**Section III- Knowledge of nursing documentation among nurses working in public hospitals in Wolaita Zone, Southern Ethiopia, 2022**

***Instruction: -Please read the following questions carefully and put (√) on the correct answer option.***

| **Items** | **Yes** | **No** | **Remark** |
| --- | --- | --- | --- |
| Documentation of patients’ care is part of professional responsibilities. |  |  |  |
| Error-free, complete, easily readable, and chronological documentation are some of the principles needed to be followed while documenting nursing care activities. |  |  |  |
| Patient care documentation is helpful to improve the quality of care, for better communication with health care staff, for education and research, and for legal protection and health planning. |  |  |  |
| Assessment data, the progress of patients, transfer and discharge of patients, care provided, and evaluation of outcomes are the main nursing activities a nurse is expected to document. |  |  |  |
| Inadequate documentation of nursing care can lead to severe injury or the death of a client and poor development of the nursing profession. |  |  |  |
| Using non-standard abbreviations when documenting patient care can lead to errors, waste of time, and confusion. |  |  |  |
| The nurse should adhere to an operational standard for nursing care documentation when documenting care provided for a patient. |  |  |  |
| Components of documenting medication administration include the names of medications, date and time of medications administered, routes and dosage of medications administered, and the nurse's name and signature. |  |  |  |
| The same nurse who provided the care or a colleague who assisted with the care is responsible for documenting the care provided to a patient. |  |  |  |
| Documenting the date and time of care, recording only what the nurse saw or did, recording in chronological order, putting single lines, making corrections clearly, and recording frequently are the main actions that protect a nurse from legal suit. |  |  |  |

**Section IV- Attitude of nursing documentation among nurses working in public hospitals in Wolaita Zone, Southern Ethiopia, 2022**

***Instruction-: There are statements about attitude of nursing documentation among nurses and each statement has five alternatives with a five-point scale. 1= strongly disagree, 2= Disagree, 3= neither agree nor disagree (neutral), 4= Agree, 5= Strongly Agree***

| **Statement** | **Strongly disagree** | **Disagree** | **Neutral** | **Agree** | **Strongly agree** |
| --- | --- | --- | --- | --- | --- |
| Nursing documentation helps to create good nurse-to-patient relationships | 1 | 2 | 3 | 4 | 5 |
| Quality documentation of nursing care can add value to my hospital | 1 | 2 | 3 | 4 | 5 |
| Proper documentation has a positive impact on patient safety | 1 | 2 | 3 | 4 | 5 |
| Although challenges are known to exist, I am expected to do complete and accurate documentation | 1 | 2 | 3 | 4 | 5 |
| A well-written report can replace an oral shift report | 1 | 2 | 3 | 4 | 5 |
| Documented care is just as important as the actual care | 1 | 2 | 3 | 4 | 5 |
| Nursing notes are meaningful and give me legal protection | 1 | 2 | 3 | 4 | 5 |
| Patients should know what we are documenting in their chart | 1 | 2 | 3 | 4 | 5 |
| Nurses have sufficient knowledge of the documentation procedure | 1 | 2 | 3 | 4 | 5 |
| Nursing admission assessment should be completed within 1 hour | 1 | 2 | 3 | 4 | 5 |

**Section V- Organizational factors affecting the practice of documentation among nurses working in public hospitals in Wolaita Zone, Southern Ethiopia, 2022**

| **SN** | **Items** | **Response** | **Remark** |
| --- | --- | --- | --- |
| 1 | Have you received any in-service training about nursing care documentation? | 1. Yes 2. No |  |
| 2 | Do you have enough time to document the nursing care provided for clients? | 1. Yes 2. No |  |
| 3 | Is there an operational standard for nursing documentation in your hospital? | 1. Yes 2. No |  |
| 4 | Are you familiar with the operational standard of nursing care? | 1. Yes 2. No |  |
| 5 | Availability of nursing care sheet for documentation | 1. Yes 2. No |  |
| 6 | Have you received any motivation from the supervisor of your hospital? | 1. Yes 2. No |  |
| 7 | Is there any obligation and follow-up to document nursing care activities from your hospital? | 1. Yes 2. No |  |
| 8 | Is there a monitoring and evaluation (M&E) system for nursing documentation in your hospital? | 1. Yes 2. No |  |
| 9 | What is the average number of patients cared for by you per shift? | 1. ≤ 2 in ICU or ≤ 6 in other than ICU 2. >2 in ICU or >6 in other than ICU |  |
| 10 | Do you feel any fatigue or exhaustion during patient care that prevents you from documenting nursing care activities? | 1. Yes 2. No |  |

**Thank You!**
